# Changing Temperature Conditions during Somatic Embryo Maturation Result in *Pinus pinaster* Plants with Altered Response to Heat Stress

**DOI:** 10.3390/ijms23031318

**Published:** 2022-01-24

**Authors:** Ester Sales, Eva Cañizares, Catia Pereira, María Amparo Pérez-Oliver, Sergio G. Nebauer, Iva Pavlović, Ondřej Novák, Juan Segura, Isabel Arrillaga

**Affiliations:** 1 Agrarian and Environmental Sciences Department, Institute for Research on Environmental Sciences (IUCA), High Polytechnic School, University of Zaragoza, Ctra. Cuarte s/n, 22071 Huesca, Spain; esalesc@unizar.es; 2 Plant Biology Department, Biotechnology and Biomedicine (BiotecMed) Institute, Universitat de València, Vicent Andrés Estellés s/n, 46100 Burjassot, Spain; ecara2@alumni.uv.es (E.C.); mapeo5@uv.es (M.A.P.-O.); juan.segura@uv.es (J.S.); 3 Department of Life Sciences, Universidade de Coimbra, 3000-456 Coimbra, Portugal; catia.pereira@student.uc.pt; 4 Department of Forestry Science, NEIKER, 01192 Arkaute, Spain; 5 Departamento de Producción Vegetal, Universitat Politècnica de València, Camino de Vera s/n, 46022 Valencia, Spain; sergonne@bvg.upv.es; 6 Laboratory of Growth Regulators, Faculty of Science of Palacký University, Institute of Experimental Botany of the Czech Academy of Sciences, Šlechtitelů 27, 78371 Olomouc, Czech Republic; iva.pavlovic@upol.cz (I.P.); ondrej.novak@upol.cz (O.N.)

**Keywords:** somatic embryogenesis, abiotic stress, resilience, photosynthesis, maritime pine, leaf anatomy

## Abstract

Under the global warming scenario, obtaining plant material with improved tolerance to abiotic stresses is a challenge for afforestation programs. In this work, maritime pine (*Pinus pinaster* Aiton) plants were produced from somatic embryos matured at different temperatures (18, 23, or 28 °C, named after M18, M23, and M28, respectively) and after 2 years in the greenhouse a heat stress treatment (45 °C for 3 h/day for 10 days) was applied. Temperature variation during embryo development resulted in altered phenotypes (leaf histology, proline content, photosynthetic rates, and hormone profile) before and after stress. The thickness of chlorenchyma was initially larger in M28 plants, but was significantly reduced after heat stress, while increased in M18 plants. Irrespective of their origin, when these plants were subjected to a heat treatment, relative water content (RWC) and photosynthetic carbon assimilation rates were not significantly affected, although M18 plants increased net photosynthesis rate after 10 days recovery (tR). M18 plants showed proline contents that increased dramatically (2.4-fold) when subjected to heat stress, while proline contents remained unaffected in M23 and M28 plants. Heat stress significantly increased abscisic acid (ABA) content in the needles of maritime pine plants (1.4-, 3.6- and 1.9-fold in M18, M23, and M28 plants, respectively), while indole-3-acetic acid content only increased in needles from M23 plants. After the heat treatment, the total cytokinin contents of needles decreased significantly, particularly in M18 and M28 plants, although levels of active forms (cytokinin bases) did not change in M18 plants. In conclusion, our results suggest that maturation of maritime pine somatic embryos at lower temperature resulted in plants with better performance when subjected to subsequent high temperature stress, as demonstrated by faster and higher proline increase, lower increases in ABA levels, no reduction in active cytokinin, and a better net photosynthesis rate recovery.

## 1. Introduction

Maritime pine (*Pinus pinaster* Aiton) is the most abundant conifer in the Mediterranean basin, showing a surprisingly ample ecological niche that covers more than 4 million ha. This species grows from mild environments in the Atlantic to harsh, dry Mediterranean climates, and from sea level to more than 2000 m in the southern regions [1]. As a consequence, large genetic variation in adaptive traits has been found between provenances for this plant species, which has an important ecological and economic value [2,3,4]. Accordingly, maritime pine is used as a model species for the study of conifer adaptation to abiotic stress [5,6]. For instance, maritime pine is the most drought-tolerant *Pinus* species in Southern Europe forests, where a combination of high temperatures and droughts has been linked to a decrease in tree growth driving forest mortality [7,8]. These adverse conditions are expected to be aggravated in the near future under the global warming scenario, therefore obtaining plant material with improved tolerance to heat and hydric stresses is a challenge for afforestation programs in this region.

Breeding of forest species by conventional techniques is hampered by the large size of the individuals and their long-generation time. In addition, traits related to abiotic stress tolerance are regulated by multiple loci that are widespread across the whole genome [9]. Biotechnological tools, such as somatic embryogenesis, are alternative approaches for producing superior individuals in conifers [10]. Somatic embryogenesis (SE) is a vegetative propagation technology that can be used to induce multiple embryos from somatic cells, and that allows the implementation of deployment strategies for improved reforestation materials [11]. One of these strategies, known as “priming,” is the induction of adaptive responses by exposing plant material at early embryogenic stages to stressful conditions, which results in an epigenetic memory that makes plants more tolerant to future stress exposure [12]. This memory is mediated by mechanisms such as sustained alterations in gene expression, changes in hormonal profiles, and accumulation of signaling proteins, and transcription factors [13]. Evidence of partly epigenetic regulation of some traits, such as the timing of bud set and active growth cessation, that were affected by environmental conditions (temperature and photoperiod) during embryo development [14], provided a way to modify cell lines by adjusting the temperature during SE processes. This induced variation is added to the unintentional selection among cell lines due to genotypical differences in the ability to perform through the process that operates in all SE stages (initiation, proliferation, and maturation), as well as in germination, plant development, and acclimatization steps, affecting overall plant yield [15].

Efficient SE protocols have been developed for commercial pine species [11], and in particular to maritime pine materials from different Spanish provenances [6,16,17]. In addition, we have recently reported that a short-time high-temperature priming applied to megagametophytes induced heat tolerance in SE-derived maritime pine plants [18]. The high temperatures used for priming in these experiments (up to 50 °C) affected the efficiency of the SE process, as occurred when milder temperatures were employed along the different steps of the protocol. Thus, increasing the temperature from 23 to 28 °C during the induction and proliferation phases of *P. pinaster* somatic embryogenesis resulted in higher rates of embryo production [6]. In *Pinus radiata*, temperature conditions at the induction step also affected the production of somatic embryos [19], whereas priming with high temperatures during the maturation phase in *P. halepensis* and *P. radiata* did not affect the number of embryos obtained; however, the regenerated plants showed better performance under drought stress [20].

Heat stress in tree species not only affects plant morphology, but also alters leaf anatomy, photosynthesis, dark respiration, stomatal conductance, and transpiration, among others [21]. At cellular and subcellular levels, the reduction in water content caused by heat negatively influences cell division and growth, as well as the functioning of photosystem II and rubisco activity. Other deleterious effects are protein denaturation, enzyme inactivation, or membrane dysfunction [22]. In response to stress, plants could alter their metabolisms, particularly by producing compatible solutes, such as proline, that are able to maintain cell turgor by osmotic adjustment. Furthermore, proline has additional roles, such as stabilizing proteins, membranes, and subcellular structures, protecting cellular functions by scavenging ROS, and acting as a signalling molecule [23]. Thus, proline plays an important role in maintaining the metabolism and growth of plants under abiotic stress conditions and many reports indicate a positive relationship between proline accumulation and tolerance of plants to various abiotic stresses [24].

Plant hormones involved in heat stress response include ABA, brassinosteroids, CKs, salicylic acid, jasmonic acid, and ethylene. Abscisic acid (ABA) is the main regulator of abiotic stress tolerance in plants. ABA signaling can be very fast without involving transcriptional activity. A good example is the control of stomatal aperture by this hormone through the biochemical regulation of ion and water transport processes. In addition, ABA regulates biomass allocation in woody plants, mainly affecting the leaf area [25]. Heat stress studies of many other plants have recently focused on physiological and molecular changes, while little is known about how heat stress affects anatomical structures [26]. 

The effects of various hormones on plant stress tolerance have been characterized in many plant species, but the results are often inconsistent. This could be due to variations of plant species and cultivars, growth stages, stress intensity and duration, and possible interactions among hormones [27]. Priming for improving thermotolerance in *Pinus* spp. by short heat treatments during SE induction affected DNA methylation and changed the expression pattern of stress-related genes in *Pinus halepensis* [28] and in *P. radiata* [29], and also altered gene expression patterns and the hormonal profile in *P. pinaster* plants [18]. Temperature during induction also had significant effects on the cytokinin (CK) profile of embryogenic lines of *Pinus halepensis* [30] and *P. radiata* [31]. Furthermore, CKs were involved in the drought tolerance of radiata pine plants derived from primed somatic embryos [32]. Temperature during maturation also affected the CK and auxin profiles of radiata pine somatic embryos [33], and thermotolerance acquisition was related to hormone profile, photosynthetic pigments, and carbohydrate contents [34,35]. Kolb and Robberecht [36] reported that the survival of seedlings of *Pinus ponderosa* in high temperatures and droughts was mediated by higher stomatal conductance and transpiration rates.

In this work, we studied the response to heat stress of maritime pine plants derived from somatic embryos induced and proliferated at 28 °C, but matured at either 18, 23, or 28 °C. Our aim was to characterize histological and physiological changes induced in SE- derived maritime pine plants by exposure to low or high temperatures during the maturation phase of the propagation protocol, and to analyze whether these changes play a role in plant adaptation to high temperature conditions.

## 2. Results

### 2.1. Plant Height and Histological Determinations in Maritime Pine Needles

Variation in maturation temperature during maritime pine somatic embryo production affected plant development, in such a way that M23 plants were on average higher than M18 and M28 plants (11.9 ± 2.2 cm front to 8.8 ± 2.6 and 9.1 ± 2.4 cm, respectively; *p* < 0.001).

**Figure 1:**
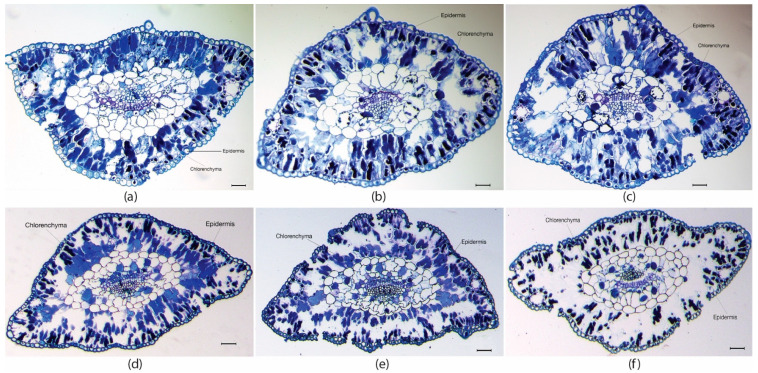
Transversal sections of needles from maritime pine plants derived from somatic embryos matured at 18 °C (**a**), 23 °C (**b**), or 28 °C (**c**) before (**a**, **b,** and **c**, respectively) or after (**d**, **e** and **f**, respectively) a heat treatment for 10 days at 45 °C for 3 h/day. Note epidermis and chlorenchymatic tissues. Bar, 50 µm.

Histological analysis also demonstrated that needles from M28 plants where thicker than those from M23 and M18 plants (Table 1 and Figure 1a–c). Nevertheless, after heat treatment (t10), needle thickness was reduced in M28 but increased in M18 and M23 plants (Table 1 and Figure 1d–f). When focusing on specific tissues, we found that the epidermis cell layer in needles from M18 plants (Figure 1a) was significantly thicker (*p* = 0.018) than in M23 (Figure 1b) and M28 plants (Figure 1c). After heat stress, the thickness of this tissue was significantly reduced in M18 plants, as well as in needles from M28 plants (Figure 1d,f; Table 1; *p* = 0.045 and *p* < 0.001, respectively). In contrast, needles from plants derived from the SE process performed in standard temperature conditions (M23 plants) did not show this histological variation when submitted to heat stress (Figure 1b,e).

Significant differences in thickness of chlorenchyma were also observed in transversal sections of needles sampled in maritime pine plants derived from somatic embryos matured at different temperatures. In M28 plants, this leaf tissue was initially larger than in M18 and M23 plants (Table 1; Figure 1a–c). At the end of the heat stress treatment, the thickness of chlorenchyma was significantly reduced in M28 plants, but increased in M18 and M23 plants (Table 1 and Figure 1d–f).

### 2.2. Water Relations and Osmotic Adjustment

The relative water content (RWC) of P. pinaster needles was similar in plants derived from SE matured at different temperatures (*p* = 0.152) and was not affected by the 10-day heat treatment (*p* = 0.260), with a mean value of 74.0% ± 5.0% (Supplementary Figure S1).

**Figure 2:**
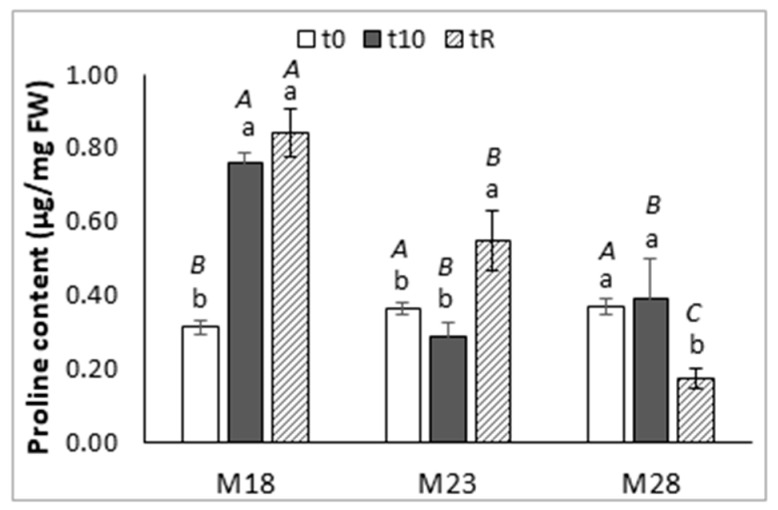
Proline content in needles of maritime pine plants derived from somatic embryos matured at different temperatures (18, 23, or 28 °C) that were employed in a heat stress experiment for 10 days at 45 °C for 3 h/day. Plants were sampled at the beginning (t0) and at the end (t10) of the stress treatment, and after a recovery period of a further 10 days (tR). Data are the mean ± SD of three replicates. Within each group of plants, values followed by the same lowercase letter did not differ significantly according to the Tukey-b test, while capital letters indicate mean separation among groups of plants sampled at the same time.

The proline content of needles varied among groups of maritime pine plants (Figure 2), and along the heat stress experiment. The basal levels of this amino acid were significantly lower in M18 than in M23 and M28 plants (*p* = 0.019), but at the end of the high temperature treatment (t10), the plants derived from somatic embryos matured at 18 °C showed significantly higher levels of this amino acid than did the M23 and M28 plants (0.76 ± 0.03 front to 0.29 ± 0.03 and 0.39 ± 0.11µg/g FW, respectively; *p* < 0.001). The great increase (2.4-fold) observed in M18 plants was maintained after the recovery time (*p* < 0.001), while in M23 and M28 plants the proline content did not change after the stress, and finally increased at tR in M23 plants (1.5-fold, *p* = 0.002) but decreased significantly in M28 plants (*p* = 0.014).

### 2.3. Photosynthesiselated Parameters

Several parameters related to photosynthesis (A_N_, net photosynthetic CO_2_ assimilation, g_s_, stomatal conductance, and C_i_ substomatal CO_2_) were measured at three point-times during the experiment: before the heat treatment (t0), at the end of the heat treatment (t10), and after the recovery period (tR).

Maritime pine M18 plants showed, on average, lower photosynthetic rates than M23 and M28 plants (*p* = 0.008); in particular, the initial photosynthetic rates in these plants were significantly lower than those determined in plants obtained under standard temperature conditions (7.4 ± 2.4 front to 10.9 ± 3.8 μmol CO_2_ m^−2^ s^−1^, *p* = 0.037). Although this parameter was slightly reduced after stress (t10 samples) in the three groups of plants, heat treatment did not have a significant effect on net photosynthetic CO_2_ assimilation, and initial levels were regained in tR samples (Table 2). Similarly, heat stress reduced stomatal conductance, but the effect was significant only in M28 plants (*p* = 0.032). Levels of substomatal CO_2_ concentration (C_i_) were significantly reduced in tR samples from M18 (*p* = 0.034) and M28 (*p* = 0.024) plants, but not in needles from M23 plants (Table 2). Higher photosynthetic rates and C_i_ concentration were significantly correlated to higher stomatal conductance (Spearman coefficient of correlation ρ = 0.466, and ρ = 0.698, respectively, *p* < 0.001). Furthermore, C_i_ decreased with stomatal conductance by the effect of heat stress, suggesting stomatal limitations to net photosynthetic rates.

### 2.4. Hormone Content

The levels of three hormones, ABA, IAA, and CKs, were also determined in maritime pine needles, before and after the heat stress treatment.

**Figure 3:**
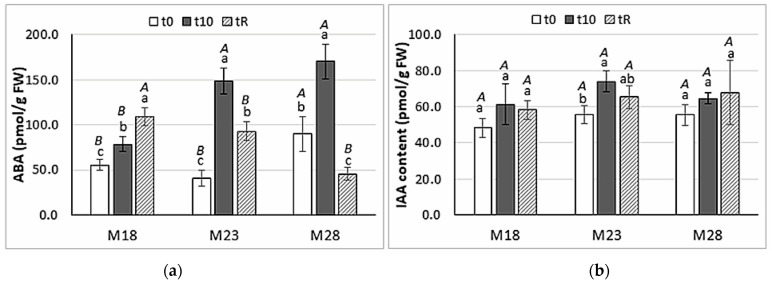
Abscisic acid (**a**) and indole-acetic acid (**b**) contents in needles of maritime pine plants derived from somatic embryos matured at different temperatures (18, 23, or 28 °C) that were employed in a heat stress experiment for 45 °C for 3 h/day, for 10 days. Plants were sampled at the beginning of the stress treatment (t0), at the end of the stress treatment (t10), and after a recovery period of a further 10 days (tR). Data are the mean ± SD of four replicates. Within each group of plants, values followed by the same lowercase letter did not differ significantly according to the Tukey-b test, while capital letters indicate mean separation among groups of plants sampled at the same time.

Needles sampled in maritime pine plants derived from SE matured at 28 °C (M28) showed basal levels of ABA (t0) significantly higher than those from M18 and M23 plants (90.1 ± 19.2 pmol/g FW front to 55.5 ± 5.7 and 41.0 ± 8.7 pmol/g FW, respectively). Heat stress treatment significantly increased ABA levels in the three groups of plants (*p* < 0.001), particularly in those derived from SE matured in standard conditions (M23), which showed a 3.6-fold increase as compared to the basal ABA level (Figure 3a). After the recovery period, M18 plants showed ABA levels higher than those observed at the end of the stress, while ABA content decreased significantly in M23 and M28 plants. It is interesting to note that basal ABA levels were not recovered in M23 plants, while in M28 plants they were lower at tR than at t0. In contrast, levels of indole-3-acetic acid (IAA) (Figure 3b) did not vary throughout the heat stress experiment in M18 (*p* = 0.097) nor in M28 plants (*p* = 0.307), while the content of this hormone significantly increased in M23 plants (*p* = 0.004) at the end of the stress (t10).

**Figure 4:**
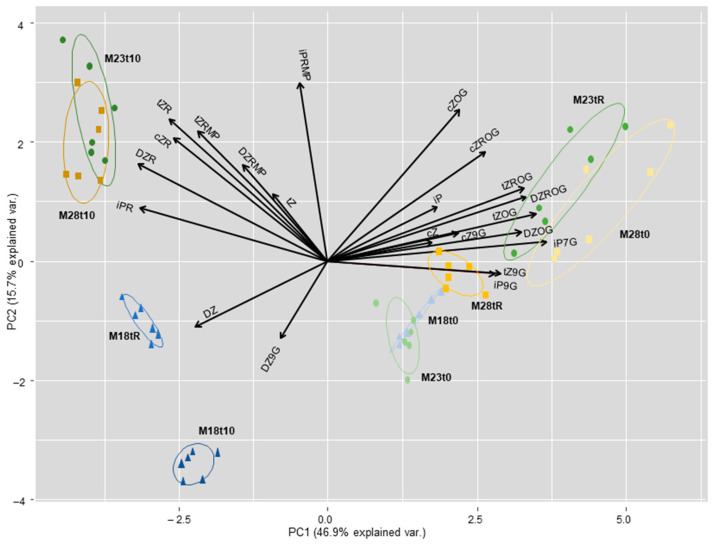
Results of the analysis when determined metabolites were grouped by CK types. Total iP-type CK content varied significantly throughout the heat stress experiment (*p* < 0.001), and this variation depended on the group of plants, as total iP levels decreased in M18 plants at the end of the stress (from 0.34 ± 0.04 to 0.22 ± 0.03 pmol/g FW), while it increased in M23 and M28 plants (from 0.26 ± 0.04 to 0.45 ± 0.06 pmol/g FW, and from 0.28 ± 0.06 to 0.50 ± 0.12 pmol/g FW, respectively). After the recovery period, total iP contents in M18 and M28 plants returned to levels similar to those at t0, while remaining unchanged in M23 plants.

Analyses of maritime pine needles determined 22 cytokinins (Supplementary Table S1) that varied among groups of plants derived from somatic embryos matured at different temperatures, and also throughout the heat stress experiment, as demonstrated by the PCA analysis (Figure 4). Basal levels of reversible CK metabolites, *O*-glucosides of *trans-*/*cis*-zeatin (*t*Z/*c*Z) and *trans-*/*cis*-zeatin ribosides, were higher in M28 plants than in M18 and M23 plants. Heat stress induced the accumulation of CKs ribosides in M23 and M28 plants, while needles of M18 plants accumulated dihydro-zeatin and the irreversible metabolite dihydrozeatin-9-glucoside. After recovery, M23 plants showed *O*-glucosides levels similar to those observed in M28 plants before the stress treatment, while M18 plants showed a different pattern of CKs.

In M18 plants, the total level of *c*Z- and *t*Z-type CKs were also significantly reduced after the stress treatment (t10), and raised again after the recovery period, while the total content of DZ types decreased significantly at this time (Table 3). These results contrasted with those observed in M23 plants, in which the total content of DZ-type CKs was significantly lower at t0 than in M18 and in M28 plants, and increased significantly in tR samples, as occurred with levels of *c*Z- and *t*Z-type CKs. In M28 plants, basal levels of *c*Z- and *t*Z-type cytokinins were significantly higher than in M18 and M23 plants (6.81 ± 1.14 pmol/g FW front to 2.65 ± 0.16 and 2.26 ± 0.06 pmol/g FW, *p* = 0.001, and 3.73 ± 0.62 pmol/g FW front to 1.45 ± 0.08 and 1.38 ± 0.05 pmol/g FW, *p* = 0.002; respectively). Both groups of compounds were significantly reduced by the heat stress treatment (t10), while the contents were partially recovered in tR samples (Table 3).

Table 4 summarizes the effect of the treatments on CKs grouped by biological form. Active CK levels were, on average, significantly higher in M18 than in M23 plants (0.17 ± 0.01 front to 0.14 ± 0.03 pmol/g FW, *p* = 0.040), although they did not differ significantly from the mean content (0.16 ± 0.06 pmol/g FW) determined in M28 plants. Furthermore, this higher level of CK bases in needles of M18 plants did not change during the heat stress experiment (*p* = 0.479), while it increased significantly after the recovery period in M23 plants (*p* = 0.007) and dropped drastically in M28 plants (*p* = 0.002). Major CK forms in the three groups of plants were *O*-glucosides (up to 96% in M28 plants after recovery); therefore, this group of metabolites drove the pattern of variation of the total CKs. In plants derived from SE performed in standard conditions (M23), heat stress slightly reduced the total CK contents, which were significantly increased after the recovery period (*p* = 0.002). In contrast, in M18 and M28 plants, the total CK levels decreased significantly after heat stress (*p* = 0.002) and these values were not fully restored after the recovery period.

## 3. Discussion

Variation in temperature conditions during the maturation step of the maritime pine SE-mediated propagation protocol resulted in modifications in the phenotype of the produced plants in terms of height, needle anatomy, and physiological and hormonal profiles, before and after a short-time heat stress treatment.

The most adequate conditions for maritime pine embryo maturation (23 °C) reported previously [6] produced higher plants after 2 years growing in a greenhouse than those derived from embryos matured at lower (18 °C) or higher temperature (28 °C); although 28 °C had been found to be the optimal temperature for the induction and proliferation stages [6]. The thicker epidermis found in M18 plants could be associated with a better adaptation to abiotic stress, as described in other species [37,38]. Exposure to high temperatures might produce foliar damage that is visible, depending on the species, the duration of exposure, and the time of year [39]. In our experiment, maritime pine plants were able to tolerate 45 °C for 3 h/day for 10 days, without any sign of visible damage at first sight. In contrast, a 30 min exposure to 48 °C produced 50% foliar damage in seedlings from *Picea glauca* [39]. Regarding anatomy, in our study, heat stress decreased chlorenchyma in M28 whereas it increased in M18 especially, which might indicate a better adaptation of these leaves to high temperatures. Similarly, palisade mesophyll was increased by heat in brassica heat-tolerant WS-1 but not in the sensitive WS-6 genotype [40]; in addition, a correlation between mesophyll cells damaged by heat stress and tolerance has been reported in salvia [41] and rhododendron [26].

Heat stress significantly affects the photosynthetic machinery and the chlorophyll and pigments biosynthetic pathways, thus disturbing the overall morpho-physiology of higher plants [42]. Heat sensitivity is intrinsically linked to drought avoidance; plants can either cool through transpiration, thus risking drought stress, or speedily close stomata to avoid drought stress while risking heat damage [43,44]. Plant responses to abnormal or extreme temperature changes are primarily mediated by hormones that might activate ROS production [45]. Heat tolerance is acquired when, in response to the excessive production of ROS, plants initiate heat stress responsive pathways for ROS-scavenging, such as the accumulation of proline and other osmolytes. ABA, the hormone regulating stomatal aperture under heat stress, has been suggested to be responsible for the initiation of other physiological responses, such as the accumulation of proline in stressed plants [24,27]. Proline has diverse roles, such as stabilizing proteins, membranes, and subcellular structures, protecting cellular functions by scavenging ROS, and acting as a signaling molecule [23]. Accumulation of such solutes may enhance stress tolerance via maintaining the cellular redox potential by lowering the generation of ROS at values suitable for metabolism when stomata are closed during osmotic stress, which limits carbon uptake and subsequently decreases NADPH consumption by the Calvin cycle [23]. This could explain why, in our abiotic stress experiment, neither RWC (Supplementary Figure S1) nor the photosynthesis-related parameters (Table 2) were significantly affected, since we only observed a reduction in stomatal conductance in needles from M28 plants sampled after the recovery period, in which a significant reduction in proline content was also detected. Needles sampled in M18 plants at t0 contained significantly lower levels of proline than M23 and M28 plants. Moreover, basal photosynthesis rates were significantly lower in M18 plants than in M23 plants and this parameter was significantly correlated with stomatal conductance (ρ = 0.493, *p* = 0.038), and inversely correlated with substomatal CO_2_ concentration (ρ = −0.474, *p* = 0.047). Heat stress treatment significantly increased the proline contents in M18 plants, and these higher contents were maintained after the recovery period, which might explain the increased net photosynthesis rate at tR in these M18 plants. This protective effect of proline was also suggested by [46] to explain the stability of the relative electron transfer rate under heat stress in seedlings of *Pinus halepensis*. Proline was also thought to mediate in alleviating the adverse effects of heat stress on photosynthesis in wheat [47] and pepper [48], as well as in the antioxidant response under heat conditions of *Quercus suber* [49] and *Olea europaea* [50]. De Diego et al. [51] also reported high levels of proline in radiata pine after recovery of a drought stress treatment, suggesting the possible implication of this metabolite in improving tolerance against future stress situations. In a previous work, we demonstrated that heat priming (50 °C) during SE induction improved performance under heat stress in *Pinus pinaster,* since primed plants showed better osmotic adjustment and higher increases in CKs, chlorophyll, soluble sugars, and starch contents, and their photosynthesis rates were less affected [18].

The hormone profile of SE-derived maritime pine plants was affected by variation in temperature during embryo maturation. Thus, needles of M28 plants showed significantly higher contents of ABA and total CK, mainly of CK bases and glucosides, and of *c*Z- and *t*Z-types, while M18 plants accumulated mainly iP-types, and both M18 and M28 plants showed higher contents of DZ-types. Higher basal levels of ABA, IAA, and CKs were also observed in primed maritime pine plants studied in our previous work, in which short temperature treatments at 37 or 50 °C were applied during the induction phase of the SE propagation protocol [18]. Variation in the hormone profiles of plants derived from somatic embryos matured at different temperatures resulted in significant coefficients of correlation between basal levels of ABA and total CKs (ρ = 0.804, *p* = 0.002); in addition, the total CK contents correlated with the content of active CK bases (ρ = 0.719, *p* = 0.001). Hormone profiles were associated with variation in needles histology, since the thicker epidermis observed in M18 plants resulted in a significant negative coefficient of correlation between epidermis thickness and IAA content (ρ = −0.729, *p* = 0.026), and the thicker chlorenchyma observed in M28 plants resulted in significant coefficients of correlation between this parameter and ABA and the total contents of CKs (ρ = 0.683, *p* = 0.020 and ρ = 0.613, *p* = 0.026, respectively). 

As mentioned, M28 maritime pine plants, which derived from somatic embryos matured at a warmer temperature than that of the standard protocol (28 front to 23 °C), accumulated higher contents of ABA (Figure 3a) but did not increase proline levels after heat stress, while M18 plants, obtained under colder conditions, showed a high increase in the concentration of this osmolyte in response to heat stress (Figure 2). Therefore, the prolonged exposure (12 weeks) of embryogenic lines at the maturation step to higher or lower temperatures also altered the performance under heat stress of the regenerated maritime pine plants. Heat or cold priming-induced cross-tolerance is very common in plants, and often results from the synergistic co-activation of multiple stress signaling pathways, involving plant hormones, as well as ROS and transcription factors [52]. For instance, heat priming during SE induced drought tolerance in *Pinus radiata* [32] and drought priming induced heat tolerance in *Festuca arundinacea* [53]. Our results also suggest that SE maturation at low temperatures produces plants with increased resilience to further heat stress.

Cytokinins have also a positive role in plant response to high temperature. Earlier studies found that the CK contents are generally reduced by heat stress, but recent studies indicate that initially active CK contents increase and are rapidly depleted, suggesting that CKs could serve as primary modulators in temperature-sensing responses. In this sense, heat acclimation and inhibition of CK degradation positively affected heat stress tolerance of Arabidopsis [54]. In our heat stress experiment, proline and the contents of CK bases were positively correlated in t10 samples (ρ = 0.703, *p* = 0.035), but inversely correlated with total CK contents (ρ = −0.850, *p* = 0.004). After the recovery period, higher proline contents were also significantly associated with higher levels of CK bases and ABA (ρ = 0.833, *p* = 0.005 and ρ = 0.917, *p* = 0.001, respectively), suggesting heat stress adaptation.

Needles sampled after heat stress in M18 and M28 plants showed a significant reduction in total CK levels, although basal contents were regained after the recovery period (Table 4). In contrast, heat treatment did not reduce total CK content in needles from M23 plants, while these rates were significantly increased after the recovery time.

Taken together, our results show the main role of proline, ABA, and cytokinin bases in the heat-stress response of maritime pine plants. In *Pinus radiata,* heat-induced thermotolerance is mediated by ABA and SA at the first stage [34], probably due to the plant’s urgent need to regulate stomatal closure and counteract the increase in oxidative membrane damage, while proline, total sugars, IAA, and CKs seem to be more relevant in longer exposures and recovery.

In conclusion, temperature conditions during somatic embryo maturation affected the regenerated maritime pine (*Pinus pinaster*) plants at anatomical and physiological levels, resulting in altered performance under heat stress conditions. Our results suggest that those plants derived from the lower temperature (18 °C) treatment were more adapted to subsequent high temperature stress, since these plants showed faster and higher proline accumulation, increased photosynthesis after recovery, a lower increase in ABA levels, and no reduction in active CK contents. Further research is required to study other counterplayers involved in this phenotype and whether it is maintained for a long time after transferring plants to field conditions. In the meantime, we state that in vitro propagation techniques might allow the generation of plants with better adaptation to extreme conditions to broaden the pathway for obtaining maritime pine plants that could be more able to cope with the global warming challenge.

## 4. Materials and Methods

### 4.1. Plant Material

Embryogenic lines were derived from three OP mother trees (1007, 1046, and 1058) belonging to the Galician Tree Breeding Program (Conselleria do Medio Rural, Xunta de Galicia, Spain) [6]. Maritime pine plants were obtained by an SE-mediated propagation protocol described by Cano et al. [16], except that the induction and proliferation phases were performed at 28 °C, and the maturation step (12 weeks) was carried out at 18, 23, or 28 °C as described by Arrillaga et al. [6]. Plants derived from somatic embryos developed in these different conditions (M18, M23, and M28 plants) were acclimated to ex vitro conditions and cultivated in 0.5 L pots in the greenhouse (SCSIE, Central Service for Experimental Research, University of Valencia, Spain) for 2 years with a 16 h photoperiod and 200–300 W·m^−2^ irradiance. The plants were watered and fertilized weekly. Twelve plants from each group were selected, randomly distributed, and used for the heat stress experiment. Plants were watered at field capacity and subjected to an increasing linear gradient (±0.1 °C/min) of air temperature that was set from 22 to 45 °C for 4 h, using an aerothermal heather, then maintained for 3 h at this temperature before decreasing to the initial conditions (22 ± 3 °C). This gradient was repeated for 10 days. Mature needles from the maritime pine plants were sampled before the stress experiment (t0), at the end of the stress experiment (t10), and after a further recovery period of 10 days (tR). Samples were frozen in liquid nitrogen and stored at −80 °C until analysis.

### 4.2. Osmotic Adjustment Determination

Relative water content (RWC) was determined in the manner described by Escandón et al. [35], using three needle fragments (1 cm long) in each of six replicates per group of plants and sampling point. Samples’ fresh weight (FW) was registered and the needles were maintained with deionized water for 24 h in the dark at 4 °C, after which the turgid weight (TW) was recorded. Then, needles were dried at 80 °C for 72 h, and dry weight (DW) was registered. RWC was calculated by using the following equation: RWC (%) = (FW − DW)/(TW − DW) × 100.

Proline was quantified in needles as described by Ábrahám et al. [55], with some modifications, in six replicates per treatment. Approximately 150–300 mg of frozen needles were homogenized in 3% sulfosalicylic acid (5 μL/mg FW), and centrifuged at 13,000 rpm for 5 min. A mixture with 100 μL of 3% sulfosalicylic acid, 200 μL of glacial acetic acid, and 200 μL of acidic ninhydrin was added to 100 μL of the supernatant of the extract, and the resulting mixture was vortexed and incubated at 96 °C for 1 h. After that, the reaction was finished on ice for 10 min. Samples were extracted in 0.5 mL of toluene and vortexed for 20 s, and the formation of the two phases was observed. The absorbance of the chromophore-containing toluene phase was read at 520 nm (Eppendorf BioSpectrometer^®^ basic, Hamburg, Germany) using toluene as the blank reagent, and proline concentration was determined from an L-proline (Sigma-Aldrich^®^, Merck KGaA, Darmstadt, Germany) standard curve with six points (0–150 μg/mL).

### 4.3. Photosynthesis-related Parameters

Photosynthesis-related parameters were determined in needles of M18, M23, and M28 maritime pine plants sampled before (t0) and after (t10) the heat stress treatment, as well as after a recovery period of a further 10 days (tR). Determinations of net photosynthetic CO_2_ assimilation rates (μmol CO_2_ m^−2^ s^−1^), stomatal conductance (mol H_2_O m^−2^ s^−1^), and substomatal CO_2_ concentration (μmol CO_2_ mol^−1^) were performed in the morning using six plants from each group and time, by using a portable photosynthesis system LICOR 6400 (LI-COR, Lincoln, NE, USA). The system was combined with a chamber for conifers (LI 6400-05). The conditions in the measuring chamber were controlled at a flow rate of 500 mol s^−1^, a saturating PAR of 1200 μmol m^−2^ s^−1^, 400 ppm CO_2_, and 60–70% relative humidity. We estimated the percentage of variation for each parameter at t10 and tR, as compared to t0 values.

### 4.4. Hormone Content Determinations

The endogenous contents of ABA, IAA, and CKs were determined in *Pinus pinaster* needles sampled from 2-year-old M18, M23, and M28 plants, before (t0) and after (t10) the heat treatment, and after the recovery period (tR). The extraction, purification, and quantification of CK metabolites were performed as described by Svačinová et al. [56] using multi-StageTip technology based on C18, SDB-RPS, and Cation-SR sorbents (Affinisep, AttractSPETM, Petit Couronne, France). For each group of plants and time, two samples were extracted in 1 mL of modified Bieleski solution containing stable isotope-labelled internal standards (0.2 pmol of CK bases, ribosides, and *N*9- and *N*7-glucosides; 0.5 pmol of CK *O*-glucosides and nucleotides). After extraction, from each sample, three technical replicates of 300 μL were purified using microSPE columns and then eluates were evaporated to dryness.

The concentration levels of IAA and ABA were determined according to the modified method described by Šimura et al. [57]. Briefly, samples (four replicates from each group of plants and time) containing 10 mg FW were extracted in aqueous solution of 50% acetonitrile (*v*/*v*). Crude extracts were loaded onto conditioned Oasis HLB columns (30 mg/1 mL, Waters) and washed with an aqueous solution of 30% acetonitrile (*v*/*v*). Flow-through fractions containing purified analytes were collected and evaporated to dryness in a vacuum evaporator.

All samples were dissolved in 30 μL of mobile phase and then analyzed using an Acquity I-Class system (Waters, Milford, MA, USA) combined with a triple quadrupole mass spectrometer (Xevo^TM^TQ-S MS, Waters, MS Technologies, Manchester, UK). A mixture of stable isotope-labelled standards of hormones was added to validate the LC-MS/MS method, and concentration levels were calculated using the isotope dilution method. All data were processed with MassLynx V4.2 software (Waters, Milford, MA, USA).

### 4.5. Histological Analyses

Maritime pine needles sampled before and after the heat stress treatment (24 replicates for each group of plants), were fixed in Karnovsky solution and stained with toluidine blue. Transversal sections were photographed under a microscope, and we determined the mean thickness of the epidermis (8 points × 10 needles per group of plants), and of the chlorenchyma (2 measures × 24 needles), using ImageJ software.

### 4.6. Statistical Analyses

Data recorded in the different experiments were subjected to analysis of variance using SPSS software (IBM Statistics, Armonk, NY, USA). When they did not adjust to a normal distribution (Kolmogorov–Smirnoff test), significant differences were assessed using the Kruskal–Wallis analysis of variance test. Correlation among parameters was also estimated by the Spearman ρ coefficient [58].

## Figures and Tables

**Table 1:** Morphology and histology of needles from 2-year-old maritime pine plants derived from somatic embryos matured at 18, 23, or 28 °C (M18, M23, and M28 plants, respectively) that were sampled before (t0) and after (t10) a heat stress treatment for 10 days at 45 °C for 3 h/day. Data are the mean ± SD of at least 10 needles; values followed by the same letter were not significantly different according to Tukey-b (*x*) or Tamhane (*y*) tests.

Plants	Needles Thickness *^x^* (µm)		Epidermis Thickness *^y^* (µm)		Chlorenchyma Thickness *^x^* (µm)	
	t0	t10	t0	t10	t0	t10
M18	553 ± 30 b	651 ± 32 a	20.3 ± 0.9 a	18.6 ± 2.3 ab	143.0 ± 11.8 c	184.8 ± 13.6 a
M23	542 ± 42 b	657 ± 57 a	18.8 ± 1.4 a	18.9 ± 1.5 a	139.8 ± 19.0 c	159.0 ± 18.3 b
M28	635 ± 24 a	557 ± 31 b	19.1 ± 1.0 a	16.6 ± 0.8 b	166.8 ± 9.8 b	136.4 ± 8.1 c

**Table 2:** Photosynthesis-related parameters in needles of maritime pine plants derived from somatic embryos matured at different temperatures (18, 23, or 28 °C), before (t0) and after (t10) a heat stress treatment (for 10 days at 45 °C for 3 h/day), as well as after a recovery period of further 10 days (tR). Six samples were employed for determining net photosynthetic CO_2_ assimilation rates (A_N;_ µmol CO_2_ m^−2^ s^−1^), stomatal conductance (g_s_; mol H_2_O m^−2^ s^−1^), and substomatal CO_2_ concentration (C_i;_ µmol CO_2_ mol^−1^).

Parameter	Plants	t0	t10	tR
A_N_^x^	M18	7.4 ± 2.4 b	7.0 ± 4.5 (−5.2%) a	9.0 ± 2.7 (22.4%) a
	M23	10.9 ± 3.8 a	8.7 ± 2.9 (−20.5%) a	10.6 ± 2.0 (−3.2%) a
	M28	9.9 ± 2.0 a	9.1 ± 0.7 (−8.4%) a	9.5 ± 1.0 (−4.0%) a
g_s_ ^y^	M18	0.24 ± 0.06 a	0.17 ± 0.06 (−29.2%) a	0.17 ± 0.12 (−29.2%) a
	M23	0.24 ± 0.11 a	0.16 ± 0.07 (−33.3%) a	0.17 ± 0.09 (−29.2%) a
	M28	0.25 ± 0.12 a	0.19 ± 0.09 (−24.0%) ab	0.13 ± 0.01 (−48.0%) b
	M18	313 ± 31 a	294 ± 54 (−6.1%) ab	264 ± 39 (−15.4%) b
(C_i_) ^z^	M23	282 ± 12 a	270 ± 36 (−4.3%) a	255 ± 35 (−9.6%) a
	M28	292 ± 21 a	271 ± 52 (−7.2%) ab	235 ± 39 (−19.4%) b

^x^, mean separation within each sampling time according to Tukey-b test. ^y^, mean separation within each group of plants according to Kruskal-Wallis ANOVA test with Bonferroni correction for multiple comparisons. ^z^, mean separation within each group of plants according to the Tukey-b test.

**Table 3:** Cytokinin content in needles of maritime pine plants derived from somatic embryos matured at different temperatures (18, 23, or 28 °C) that were employed in a heat stress experiment for 10 days at 45 °C. Plants were sampled at the beginning of the stress treatment (t0), at the end of the stress treatment (t10), and after a recovery period of a further 10 days (tR). Data are the mean ± SE of six replicates and 22 cytokinins grouped by type: cis-zeatin (cZ), trans-zeatin (tZ), dihydro-zeatin (DZ), and isopentenyladenine (iP). Within each group of plants, values followed by the same letter did not differ significantly (*p* < 0.05), according to Kruskal-Wallis one-way ANOVA.

Plants	Time	Total iP-Types	Total tZ-Types	Total DZ-Types	Total cZ-Types
M18	t0	0.34 ± 0.04 a	1.45 ± 0.08 a	2.30 ± 0.07 a	2.65 ± 0.16 a
	t10	0.22 ± 0.03 b	0.34 ± 0.05 b	0.86 ± 0.12 ab	0.87 ± 0.08 b
	tR	0.35 ± 0.04 a	0.47 ± 0.05 ab	0.62 ± 0.03 b	1.87 ± 0.04 a
M23	t0	0.26 ± 0.04 b	1.38 ± 0.05 ab	1.56 ± 0.09 ab	2.26 ± 0.06 b
	t10	0.45 ± 0.06 a	0.95 ± 0.11 b	0.87 ± 0.15 b	2.67 ± 0.22 ab
	tR	0.45 ± 0.08 a	2.72 ± 0.52 a	4.12 ± 0.50 a	4.22 ± 0.50 a
M28	t0	0.28 ± 0.06 b	3.73 ± 0.62 a	2.44 ± 0.34 a	6.81 ± 1.14 a
	t10	0.50 ± 0.12 a	0.82 ± 0.05 b	0.64 ± 0.09 b	2.90 ± 0.28 b
	tR	0.28 ± 0.03 b	2.56 ± 0.14 a	2.19 ± 0.16 a	4.60 ± 0.32 ab

**Table 4:** Cytokinin content in needles of maritime pine plants derived from somatic embryos matured at different temperatures (18, 23, or 28 °C) that were employed in a heat stress experiment for 10 days at 45 °C for 3 h/day. Plants were sampled at the beginning of the stress treatment (t0), at the end of the stress treatment (t10), and after a recovery period of a further 10 days (tR). Data are the mean ± SE of six replicates and 22 compounds grouped by conjugated structure, and the percentages refer to total CK content.

Plants	Time	Active Forms	Transport Forms	Precursors	Reversible Metabolites	Irreversible Metabolites	Total CK ^y^
		CKs Bases ^x^	CK Ribosides ^y^	CK Nucleotides ^y^	CK O-glucosides ^y^	CK N-glucosides ^y^	
M18	t0	0.16 ± 0.03 (2.4%) a	0.06 ± 0.01 (0.9%) b	0.20 ± 0.02 (3.0%) a	6.28 ± 0.17 (93.3%) a	0.03 ± 0.00 (0.4%) a	6.73 ± 0.18 a
	t10	0.16 ± 0.03 (7.1%) a	0.13 ± 0.02 (5.9%) ab	0.09 ± 0.01 (3.0%) b	1.89 ± 0.21 (82.6) b	0.01 ± 0.00 (0.5%) ab	2.28 ± 0.26 b
	tR	0.18 ± 0.03 (5.3%) a	0.24 ± 0.03 (7.1%) a	0.16 ± 0.01 (4.8%) ab	2.74 ± 0.06 (82.6%) ab	0.01 ± 0.00 (0.2%) b	3.31 ± 0.11 ab
M23	t0	0.12 ± 0.04 (2.2%) b	0.05 ± 0.01 (0.9%) b	0.20 ± 0.04 (3.6%) b	5.07 ± 0.09 (92.8%) ab	0.03 ± 0.02 (0.5%) ab	5.46 ± 0.13 ab
	t10	0.12 ± 0.01 (2.4%) b	0.29 ± 0.03 (5.9%) a	0.59 ± 0.09 (12.0%) a	3.93 ± 0.37 (79.6%) b	0.01 ± 0.00 (0.1%) b	4.94 ± 0.47 b
	tR	0.17 ± 0.02 (1.5%) a	0.09 ± 0.00 (0.8%) ab	0.29 ± 0.07 (2.5%) ab	10.92 ± 1.48 (94.9%) a	0.04 ± 0.01 (0.3%) a	11.50± 1.55 a
M28	t0	0.21 ± 0.06 (1.6%) a	0.04 ± 0.02 (0.3%) b	0.14 ± 0.04 (1.1%) b	12.82 ± 2.04 (96.7%) a	0.04 ± 0.01 (0.3%) a	13.25 ± 2.15 a
	t10	0.17 ± 0.04 (3.5%) a	0.33 ± 0.02 (6.9%) a	0.41 ± 0.08 (8.4%) a	3.94 ± 0.42 (81.1%) b	0.01 ± 0.02 (0.1%) b	4.86 ± 0.48 b
	tR	0.09 ± 0.02 (1.0%) b	0.07 ± 0.00 (0.7%) ab	0.20 ± 0.03 (2.0%) ab	9.24 ± 0.62 (96.0%) ab	0.03 ± 0.00 (0.3%) ab	9.63 ± 0.60 ab

^x^, mean separation within each group of plants according to the Tukey-b test. ^y^, mean separation within each group of plants according to the Kruskal-Wallis test with Bonferroni correction for multiple comparisons.

## Supplementary Materials

The following supporting information can be downloaded at: https://www.mdpi.com/article/10.3390/ijms23031318/s1.
