# Supplementary material for: Changing Temperature Conditions during Somatic Embryo Maturation Result in Pinus pinaster Plants with Altered Response to Heat Stress
Source: Int J Mol Sci. 2022 Jan 24;23(3):1318. doi: 10.3390/ijms23031318 (PMC8835971; doi:10.3390/ijms23031318)
Supplement: Supplementary file 1 [file ijms-23-01318-s001.zip › ijms-1555485-supplementary.pdf]

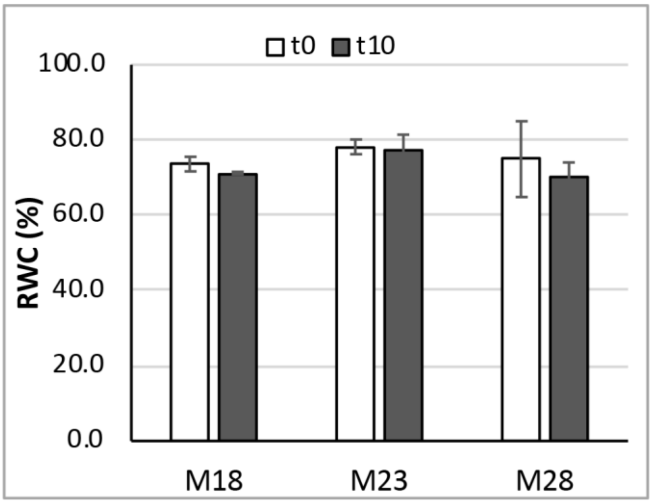

Figure S1. Relative water content (RWC) of maritime pine needles sampled in plants derived from somatic embryos matured at different temperatures (18, 23 or 28°C) that were employed in a heat stress experiment for 10 days at 45°C. Plants were sampled at the beginning (t0) and at the end (t10) of the stress treatment. Data are mean ± SD of 6 replicates.

Supplementary data

Table S1. Cytokinins determined in this study.

**Abbr.**	**Cytokinin**
De novo biosynthesis of iP-, tZ- and DHZ-type CKs	
iP	isopentenyladenine
iPR	isopentenyladenosine
iPRMP	isopentenyladenosine-5'monophosphate
iP7G	isopentenyladenine-7-glucoside
iP9G	isopentenyladenine-9-glucoside
*t*Z	*trans*-zeatin
*t*ZR	*trans*-zeatin riboside
*t*ZRMP	*trans*-zeatin riboside-5'monophosphate
*t*ZOG	*trans*-zeatin-O-glucoside
*t*ZROG	*trans*-zeatin riboside-O-glucoside
*t*Z7G	*trans*-zeatin-7-glucoside*
*t*Z9G	*trans*-zeatin-9-glucoside
DZ	dihydrozeatin
DZR	dihydrozeatin riboside
DZRMP	dihydrozeatin riboside-5'monophosphate

DZOG	dihydrozeatin-O-glucoside
DZROG	dihydrozeatin riboside-O-glucoside
DZ7G	dihydrozeatin-7-glucoside*
DZ9G	dihydrozeatin-9-glucoside
tRNA degradation pathway (main source of cZ-types	
*c*Z	*cis*-zeatin
*c*ZR	*cis*-zeatin riboside
*c*ZRMP	*cis*-zeatin riboside-5'monophosphate*
*c*ZOG	*cis*-zeatin-O-glucoside
*c*ZROG	*cis*-zeatin riboside-O-glucoside
*c*Z7G	*cis*-zeatin-7-glucoside*
*c*Z9G	*cis*-zeatin-9-glucoside

*Not detected in the analysed samples
